# Concurrent Tuberculosis and Influenza, South Korea

**DOI:** 10.3201/eid1901.111613

**Published:** 2013-01

**Authors:** Ji Yun Noh, Jacob Lee, Won Suk Choi, Joon Young Song, Yu Bin Seo, In Seon Kim, Hee Jin Cheong, Woo Joo Kim

**Affiliations:** Author affiliations: Korea University College of Medicine, Seoul, South Korea (J.Y. Noh, W.S. Choi, J.Y. Song, Y.B. Seo, I.S. Kim, H.J. Cheong, W.J. Kim);; Asia Pacific Influenza Institute, Seoul (J.Y. Noh, W.S. Choi, J.Y. Song, Y.B. Seo, I.S. Kim, H.J. Cheong, W.J. Kim);; Hallym University College of Medicine, Chuncheon, South Korea (J. Lee);; Transgovernmental Enterprise for Pandemic Influenza in Korea, Seoul (W.J. Kim)

**Keywords:** Tuberculosis, influenza, pandemics, South Korea, respiratory infections, tuberculosis and other mycobacteria, viruses

**To the Editor:** The concurrence of active pulmonary tuberculosis (TB) and influenza in immunocompetent hosts is rarely reported. Such concurrence could distract clinicians from diagnosing TB during an influenza epidemic. We describe 7 cases of concurrent active pulmonary TB and influenza A(H1N1)pdm09 virus infection in South Korea.

At 2 teaching hospitals in Seoul, medical records were reviewed retrospectively. Among the 12,196 patients for whom A(H1N1)pdm09 infection was confirmed by real-time reverse transcription PCR from May 2009 through May 2011, a total of 7 (0.06%) were co-infected with newly diagnosed active pulmonary TB (Table). Patients who had a history of TB diagnosis were excluded.

**Table:** Case summary of concurrent active pulmonary TB and influenza A(H1N1)pdm09 infection*

Patient age, y/sex	Date of influenza diagnosis	Underlying disease	Days from influenza to TB diagnosis	Lymphocyte count at initial visit, cells/µL (%)	Specimen/ auramine-rhodamine stain result	Specimen/ TB PCR result	MTB source	Radiographic findings	Days hospitalized
17/F	2009 Sep	None	17	NA	BAL/+	BAL/+	BAL	Patchy consolidation in LUL	0
15/M	2009 Sep	None	2	380 (7.3)	Sputum/+	Sputum/+	Sputum	Pneumonic infiltration in both upper lobes, cavitary lesion in LUL	7
15/F	2009 Oct	None	17	NA	Sputum/+	Sputum/−	Sputum	Nodular infiltration with cavity in LUL	10
26/M	2009 Nov	None	1	1,406 (18.5)	Sputum/+	Sputum/+	Sputum	Multiple nodules and cavities in RUL and RML	6
29/M†	2010 Feb	None	2	NA	Sputum/−, BAL/−	BAL/+	Sputum, BAL	Pneumonic infiltration in RUL	0
68/M	2010 Feb	Colon cancer	1	230 (4.9)	Sputum/+	NA	Sputum	Lobar pneumonia in RLL	10
24/F	2011 Feb	Previous wedge resection to treat pneumothorax	7	1,433 (37.7)	Sputum/−, BAL/+	Sputum/−, BAL/+	BAL	Patchy opacity in left middle lung zone	0

*All patients survived. TB, tuberculosis; MTB, *Mycobacterium tuberculosis*; NA, not available; BAL, bronchoalveolar lavage; LUL, left upper lobe; RUL, right upper lobe; RML, right middle lobe; RLL, right lower lobe. †This patient had a history of 7.5 pack-years of smoking.

Among the 7 co-infected patients, 6 (85.7%) were <30 years of age. All but 1 patient, who had colon cancer, had been previously healthy. No patients had diabetes mellitus or HIV infection. One patient was a current smoker. For 5 patients, pulmonary TB was diagnosed within 1 week from the date of influenza diagnosis; initial chest radiographic findings were suggestive of active TB or pneumonia. Another 2 patients, for whom radiographic examination was not performed at the first visit, experienced worsening cough and blood-tinged sputum after improvement of influenza; laboratory tests for TB were performed, and pulmonary TB was diagnosed 17 days after the date of influenza diagnosis. For 4 patients, computed tomography of the chest was performed, and multiple nodular lesions, cavities, and tree-in-bud appearance were found. Lymphopenia at initial visit was detected in 2 patients. All *Mycobacterium tuberculosis* isolates were sensitive to anti-TB drugs, and clinical outcomes were good for all patients.

For persons infected with *M. tuberculosis*, lifetime risk for development of active TB is 5%–10%; this risk increases for those with immunocompromising conditions (*1*). One study reported that pulmonary TB was a risk factor for A(H1N1)pdm09 infection (*2*). However, the concurrence of influenza and pulmonary TB has been reported only a few times, and the findings have been mostly descriptive and somewhat contradictory. An old report, from 1919, describes TB diagnoses for patients who were not recovered completely from influenza pneumonia (*3*). During 1957–1958, Löfgren and Callans (*4*) observed 46 patients with newly detected TB that had been diagnosed shortly after Asian influenza; among them, 4 had a history of typical influenza.

In South Africa, among 72 patients who died of A(H1N1)pdm09 infection, 7 also had active TB (*5*). In Taiwan, TB and A(H1N1)pdm09 infection in a lung cancer patient was reported (*6*). In Japan, a fatal case of influenza pneumonia combined with *Streptococcus pneumoniae* and *M. tuberculosis* infection in a patient with diabetes mellitus was reported (*7*). Although the 2 patients from Taiwan and Japan had concurrent illnesses, 6 of the 7 patients in our study had been healthy (*6*,*7*). Radiographic abnormalities for the patients reported here were similar to those reported for other patients, but more cavitary lesions were found for the patients reported here.

Although it is not clear whether influenza accelerates emergence of TB, some animal studies suggest that influenza-associated TB is possible. In mouse studies, simultaneous injection of tubercle bacilli into the peritoneum and intranasal inoculation with influenza A virus (PR8) resulted in more rapid and extensive development of pulmonary tuberculous lesions than did infection with tubercle bacilli only (*8*). In a mouse model of chronic infection with *M. bovis* BCG, acute infection with influenza virus moderately increased the load of acid-fast bacilli in the liver, although this change was not significant (*9*).

It is possible that temporary suppression of T-cell immunity by A(H1N1)pdm09 virus might alter the course of *M. tuberculosis* infection. Among influenza patients, CD4^+^ T cells were depleted and a subset of Th17 cells were preferentially lost at an early stage of infection; Th17 cells that produce proinflammatory cytokine interleukin-17 are associated with a protective immune response (*10*). Among 4 patients for whom laboratory examination was conducted at initial visit, 2 were lymphopenic. However, individual lymphocyte subsets were not checked, and a functional assay of lymphocytes was not conducted. Further studies of serial quantification and functional assay of lymphocytes at the acute stage of influenza and its effect on host susceptibility to TB in animals and humans are needed.

The concurrence of TB and influenza could be a simple overlap. In 2009, the case notification rate of pulmonary TB in South Korea was 58.2 cases per 100,000 population; in 2010, it was 56.5. However, if influenza actually amplifies TB, TB might be underestimated and missed in influenza patients. Thus, large-scale observational epidemiologic studies on the changing incidence of TB during the influenza postpandemic era are needed. Especially in TB-endemic areas, physicians should consider a concurrent pulmonary TB diagnosis for influenza patients with radiologic abnormalities consistent with TB or with prolonged respiratory symptoms.
